# Supplementary material for: Pharmacokinetics, bactericidal activity and toxicity of short oral regimens for rifampicin-resistant tuberculosis treatment
Source: Nat Commun. 2026 Jun 10;17:7830. doi: 10.1038/s41467-026-74335-y (PMC13439552; doi:10.1038/s41467-026-74335-y)
Supplement: Supplementary file 1 [file 41467_2026_74335_MOESM1_ESM.pdf]

Pharmacokinetics, bactericidal activity and toxicity of short oral regimens for rifampicin-resistant tuberculosis treatment

*Bern-Thomas Nyang'wa [1,2], Ilaria Motta [3], Ronelle Moodliar [4], Varvara Solodovnikova [5], Shakira Rajaram [6], Mohammed Rasool [6], Catherine Berry [7], David A.J. Moore [8], #Geraint Davies [9], #*Frank Kloprogge [2]

# These authors jointly supervised this work

[1] Médecins sans Frontieres, Public Health Department, Amsterdam, The Netherlands

[2] Institute for Global Health, University College London, London, United Kingdom

[3] University College London, Innovative Clinical Trials Unit, London, United Kingdom

[4] THINK (TB & HIV Investigative Network) Durban, South Africa.

[5] Republican Scientific and Practical Centre of Pulmonology and Tuberculosis, Minsk, Belarus.

[6] Clinical HIV Research Unit (CHRU), Wits Health Consortium (WHC), Department of Internal Medicine, School of Clinical medicine, Faculty of Health Sciences, University of Witwatersrand, Johannesburg, South Africa

[7] Médecins sans Frontieres, Manson Unit, Public Health Department, London, United Kingdom

[8] London School of Hygiene and Tropical Medicine, Clinical Research Department, London, United Kingdom

[9] University of Liverpool, Department of Clinical Infection, Microbiology and Immunology, Liverpool, United Kingdom

*** Corresponding authors**

Bern-Thomas Nyang'wa: bern.nyangwa@london.msf.org

Frank Kloprogge: f.kloprogge@ucl.ac.uk

Supplementary Information

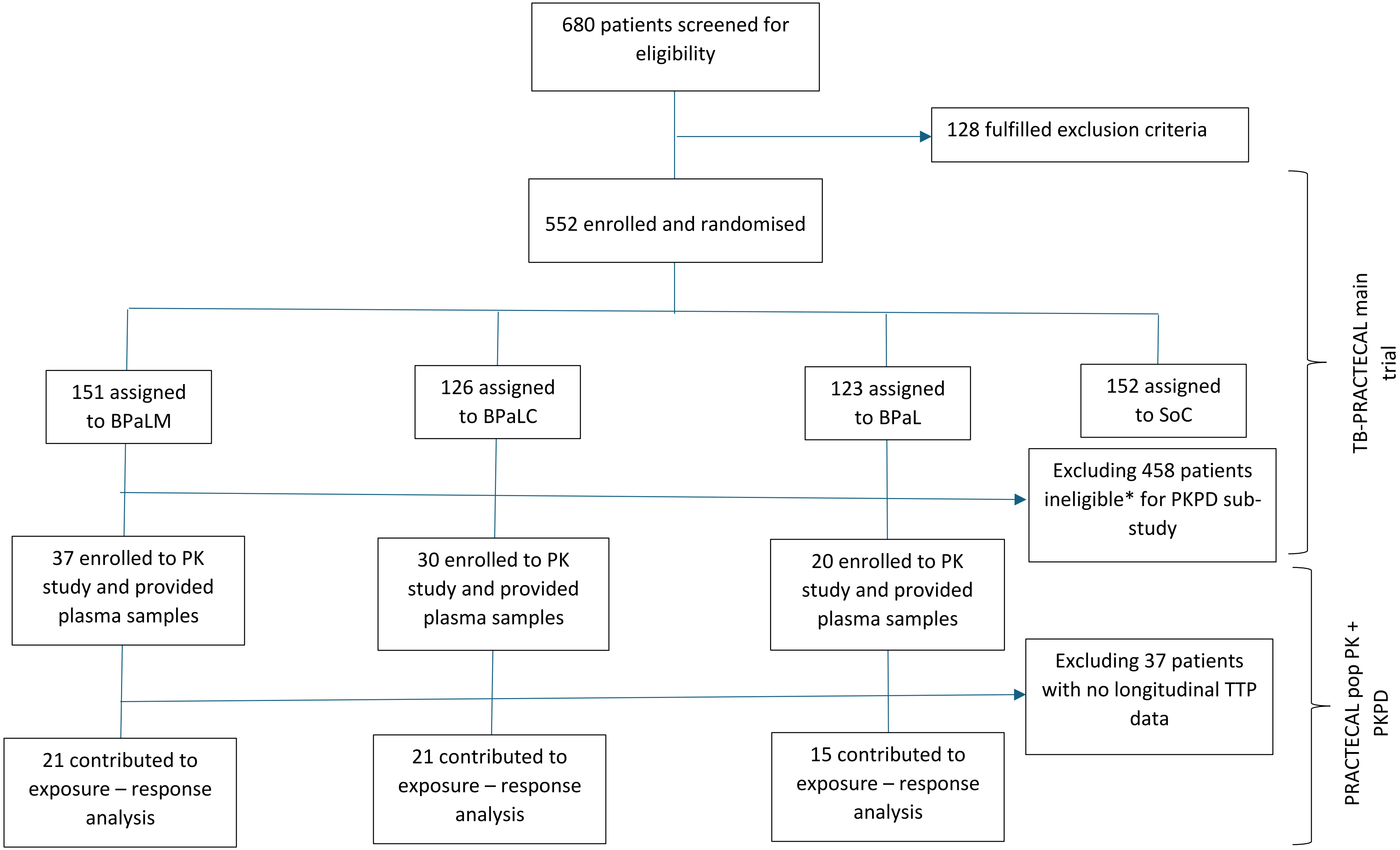

*ineligible = in SoC, at site not participating in sub-study, recruited into main trial before sub-study started

Figure S1: Study profile, a flowchart describing the path that the final analysis population per arm took through the clinical trial and sub study.

Pharmacokinetics

Table S1: Population pharmacokinetic model estimates for bedaquiline, pretomanid, linezolid, clofazimine and moxifloxacin of participants in the PRACTECAL-PKPD study.

	Bedaquiline				pretomanid				linezolid				clofazimine				moxifloxacin			
Parameter	Mean estimate (95%CI)	%RSE	BSV (CV%)	Shrink %	Mean estimate (95%CI)	%RSE	BSV (CV%)	Shrink %	Mean estimate (95%CI)	%RSE	BSV (CV%)	Shrink %	Mean estimate (95%CI)	%RSE	BSV (CV%)	Shrink %	Mean estimate (95%CI)	%RSE	BSV (CV%)	Shrink %
cl (l/h)	1.93 (1.58, 2.36)	15.6	43.1	8.06%<	3.1 (2.87, 3.33)	3.36	32.9	9.65%<	6.66 (6.04, 7.34)	2.62	28.2	11.4%<	6.84 (5.73, 8.15)	4.68	77.3	8.78%<	14.2 (12.2, 16.4)	2.84	23.1	18.8%<
vc (l)	103 (73.6, 143)	3.65	132	10.3%<	102 (81.4, 127)	2.45	33.6	36.7%>	58.8 (51.7, 66.9)	1.61	5.63	94.6%>	1.75e+03 (1.44e+03, 2.12e+03)	1.32	173	2.39%<	167 (133, 211)	2.29	11.1	27.3%=
vp (l)	6.51e+03 (5.45e+03, 7.77e+03)	1.03	37.1	16.7%<									9.15e+03 (7.79e+03, 1.07e+04)	0.895	44.6	26.1%=	87.4	FIXED		
q (l/h)	7.49 (5.58, 10.1)	7.45	66.1	15.7%<									41.7 (34.6, 50.4)	2.58	82	40.9%>	2.3	FIXED		
vp2 (l)	98.9 (49.3, 199)	7.74																		
q2 (l/h)	7.02 (4.26, 11.6)	13.1																		
prop.err	0.321				0.322								0.198							
add.err	59.5				368								16.4							
logn.err									0.888								0.645			
ka (h-1)					0.316 (0.203,	19.6			1.23	FIXED			0.67	FIXED			0.569	FIXED		
tlag (h)													0.62	FIXED						
MAT	0.66	FIXED																		
FR	0.47	FIXED																		

BSV: between patient variability, Shrink: eta-shrinkage, RSE: relative standard error, prop.err: proportional error, add.err: additive error, logn.sd additive residual variability on both-sided log-transformed data. MAT refers to fraction of time for both delay and 90% complete absorption. Allometric body size scaling was applied to clearance(cl) and volume (v) parameters based on Fat Free Mass with the power fixed to 0.75 and 1, respectively. cl: clearance, vc: central compartment volume, vp: peripheral compartment volume, q: inter-compartmental clearance

Efficacy outcomes

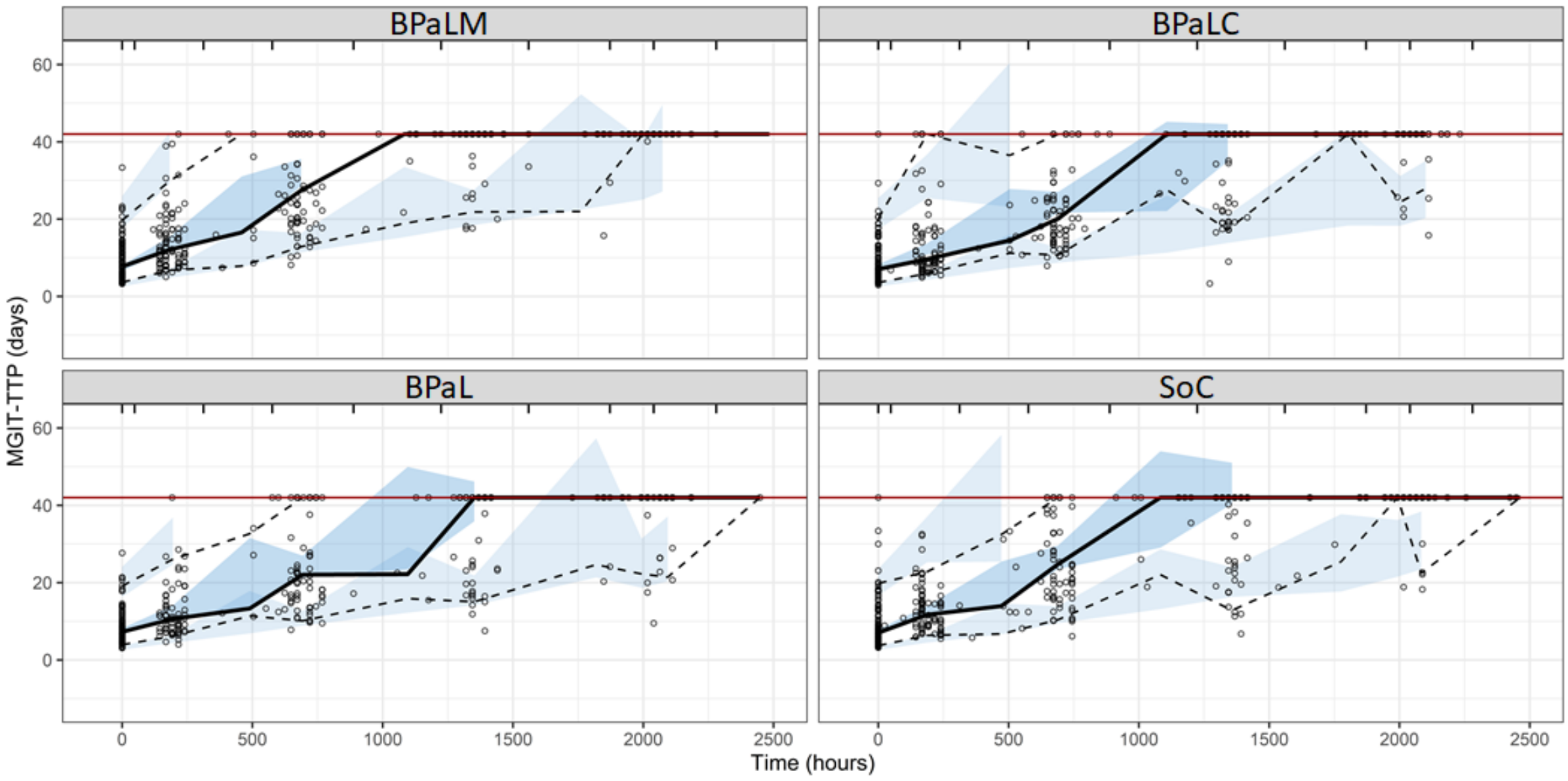

Figure S2: Visual predictive check of the model showing longitudinal MGIT-TTP in days vs time on treatment in hours, stratified by study arm. Dots represent observations, solid and dashed lines the median and 2.5-97.5 percentiles of the observe data with corresponding 90% Confidence Intervals overlaid ($n_{simulations}$ = 500). The horizontal red line represents the upper limit of quantification, i.e. 42 days). Source data are provided as model file labelled MGIT-TTP_final_model

Table S2: Population pharmacodynamic parameters for the MGIT-TTP model, describing the slope and effect of the covariates.

	**Mean estimate of slope parameters (95%CI)**	**%RSE**	**BSV (CV%)**	**Shrink (SD)%**
**MGIT-TTP vs time slope**	0.0397 (0.0341, 0.0463)	2.43	30.7	24.4%=
**MGIT-TTP intercept**	14.4 (13.2, 15.6)	1.54	19.3	33.7%>
**Residual unexplained variability**	0.393			
**Effect of smear 1+** on MGIT-TTP intercept	0.619 (0.552,0.693)	12.1		
**Effect of smear 2+** on MGIT-TTP intercept	0.431 (0.389,0.476)	6.11		
**Effect of smear 3+** on MGIT-TTP intercept	0.356 (0.319,0.398)	5.47		
**Effect of cavitation** on MGIT-TTP slope	0.728 (0.642,0.825)	20.1		
**Effect of BPaL or BPaLC** on MGIT-TTP slope	0.852 (0.734,0.988)	47.1		
**Effect of BPaLM** on MGIT-TTP slope	1.204 (1.03,1.408)	42.8		
**Effect of smear 2+ or smear 3+** on MGIT-TTP slope	0.804 (0.712,0.908)	28.5		

BSV: between patient variability, Shrink: eta-shrinkage, RSE: relative standard error.

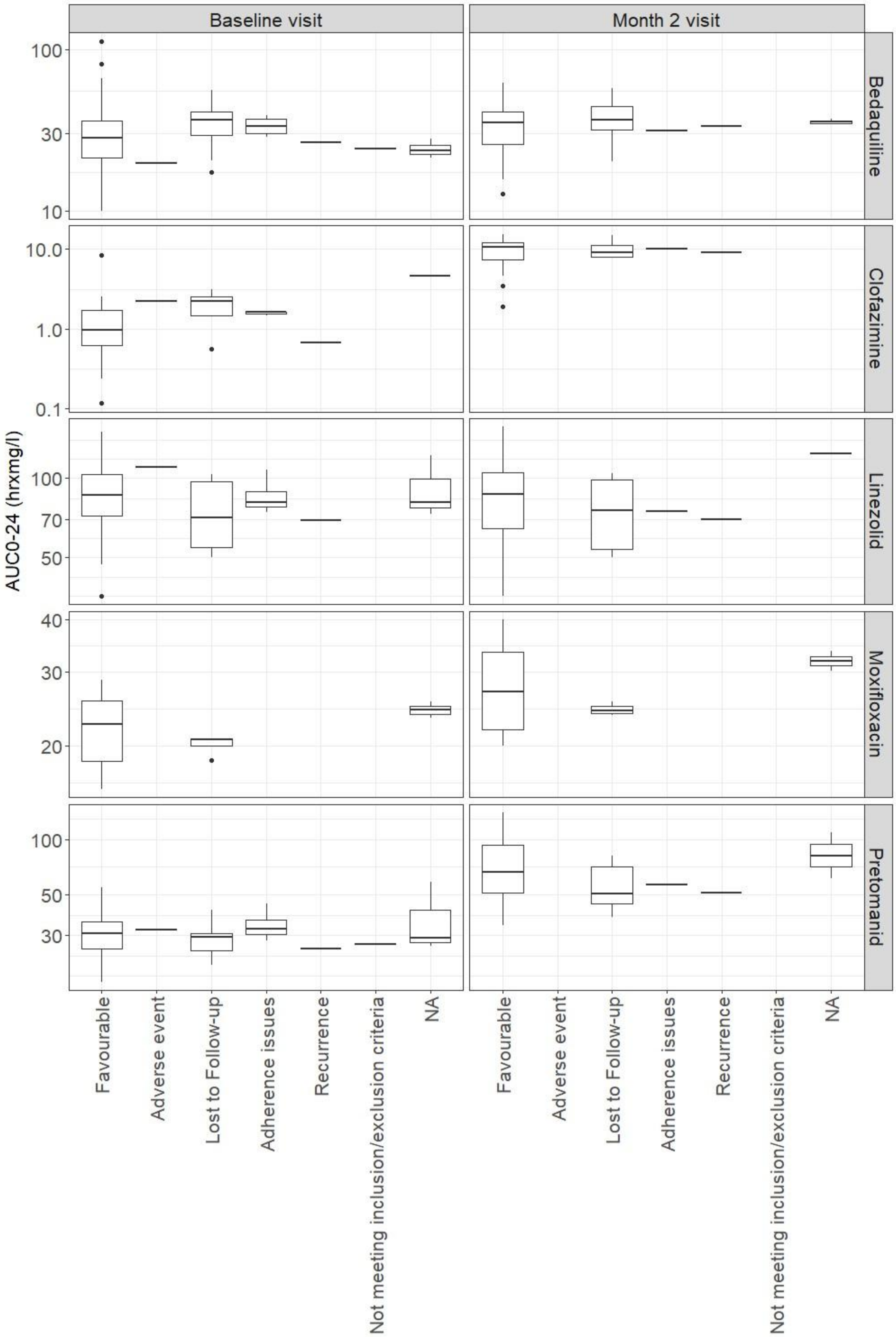

NA: Outcome not applicable as patient was excluded from the modified intention to treat population

Figure S3: box plots of antimicrobial exposure by the various unsuccessful outcome categories. bedaquiline (n=49), pretomanid (n=49), linezolid (n=42), moxifloxacin (n=19), and clofazimine (n=18). Source data are provided as a Source Data file in sheet labelled Fig S3.

Safety outcomes

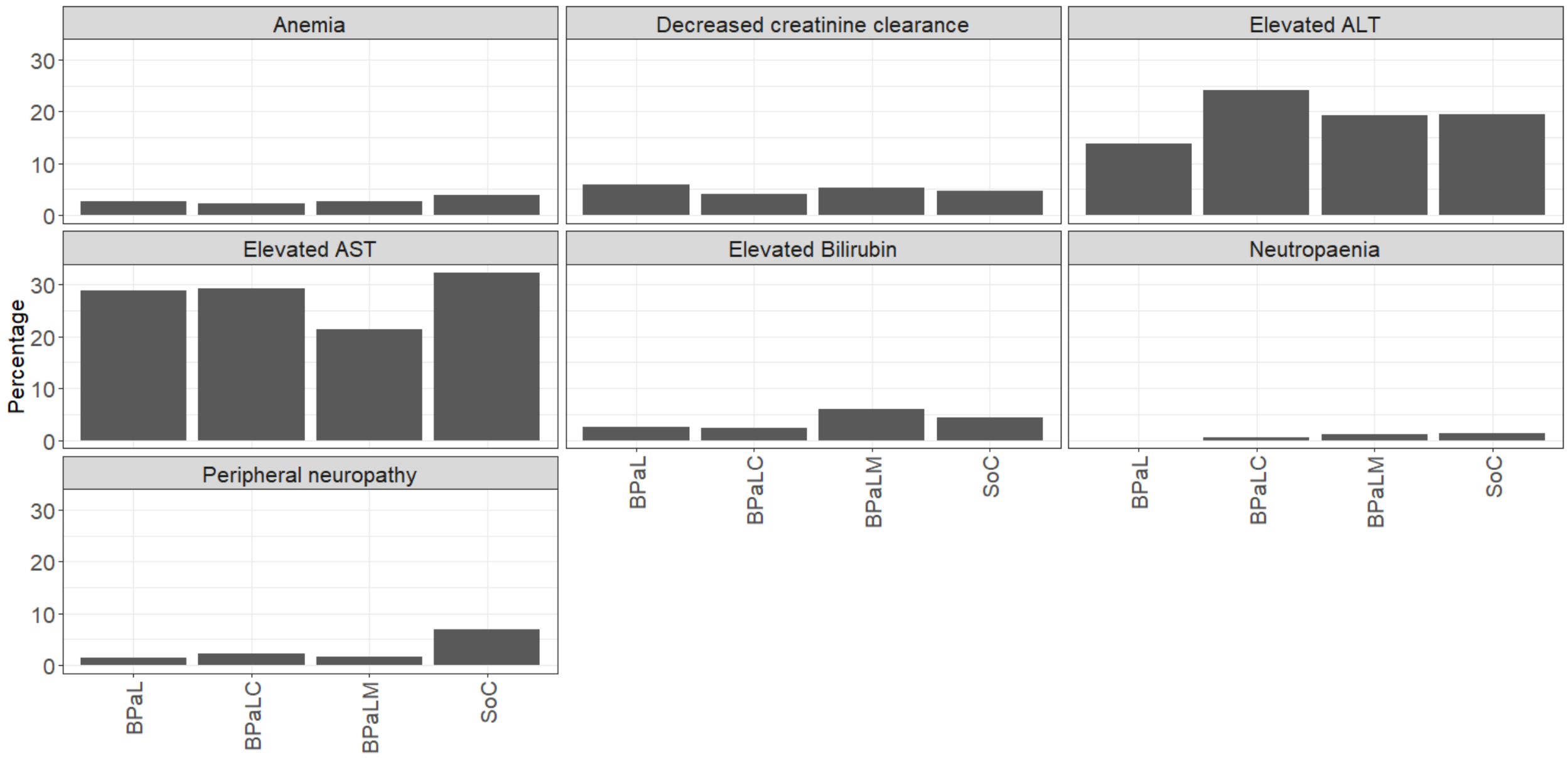

Figure S4: Visualisation of the occurrence of related adverse events per study arm, presented as the proportion of patients in the study reporting at least one adverse event (n = 514). Source data are provided as a Source Data file in sheet labelled Fig S4.
