# Supplementary material for: Pharmacokinetics, bactericidal activity and toxicity of short oral regimens for rifampicin-resistant tuberculosis treatment
Source: Nat Commun. 2026 Jun 10;17:7830. doi: 10.1038/s41467-026-74335-y (PMC13439552; doi:10.1038/s41467-026-74335-y)
Supplement: Supplementary file 2 [file 41467_2026_74335_MOESM2_ESM.pdf]

nature portfolio

Corresponding author(s): Bern-Thomas Nyang'wa

Last updated by author(s): May 22, 2026

Reporting Summary

Nature Portfolio wishes to improve the reproducibility of the work that we publish. This form provides structure for consistency and transparency in reporting. For further information on Nature Portfolio policies, see our Editorial Policies and the Editorial Policy Checklist.

Statistics

For all statistical analyses, confirm that the following items are present in the figure legend, table legend, main text, or Methods section.

n/a	Confirmed	
☐	☒	The exact sample size (n) for each experimental group/condition, given as a discrete number and unit of measurement
☐	☒	A statement on whether measurements were taken from distinct samples or whether the same sample was measured repeatedly
☐	☒	The statistical test(s) used AND whether they are one- or two-sided<br>*Only common tests should be described solely by name; describe more complex techniques in the Methods section.*
☐	☒	A description of all covariates tested
☒	☐	A description of any assumptions or corrections, such as tests of normality and adjustment for multiple comparisons
☐	☒	A full description of the statistical parameters including central tendency (e.g. means) or other basic estimates (e.g. regression coefficient) AND variation (e.g. standard deviation) or associated estimates of uncertainty (e.g. confidence intervals)
☐	☒	For null hypothesis testing, the test statistic (e.g. F, t, r) with confidence intervals, effect sizes, degrees of freedom and P value noted<br>*Give P values as exact values whenever suitable.*
☒	☐	For Bayesian analysis, information on the choice of priors and Markov chain Monte Carlo settings
☒	☐	For hierarchical and complex designs, identification of the appropriate level for tests and full reporting of outcomes
☒	☐	Estimates of effect sizes (e.g. Cohen's d, Pearson's r), indicating how they were calculated

Our web collection on statistics for biologists contains articles on many of the points above.

Software and code

Policy information about availability of computer code

Data collection	Dose intake and sample collection timing data was collected using KoboCollect v1.14.0a, clinical data was collected using OpenClinica v3.12, Bioanalytical data acquisition and integration was performed by Analyst version 1.6.1 and Multi Quant version 3.0, mycobacteriology data was collected using MGIT 960 instrument connected to an EpiCenter™ equipped with TBeXIST module (BD)
Data analysis	Analyses including nonlinear mixed-effect modelling were performed in R v4.1.2 using nlmixr2 for the population PK and pharmacodynamic model development. Clinical data analyses was conducted using Stata v15.1

For manuscripts utilizing custom algorithms or software that are central to the research but not yet described in published literature, software must be made available to editors and reviewers. We strongly encourage code deposition in a community repository (e.g. GitHub). See the Nature Portfolio guidelines for submitting code & software for further information.

Data

Policy information about availability of data

All manuscripts must include a data availability statement. This statement should provide the following information, where applicable:

- Accession codes, unique identifiers, or web links for publicly available datasets
- A description of any restrictions on data availability
- For clinical datasets or third party data, please ensure that the statement adheres to our policy

Deidentified pharmacokinetic data will be available to researchers upon a written request to the Medical Director, Médecins sans Frontières, Operational Centre Amsterdam, the Netherlands. Clinical and mycobacteriology data is available from TB-PACTS study ID 1035 at: https://c-path.org/tools-platforms/tb-pacts and

World Health Organization Tuberculosis treatment individual patient data platform, University College London. Available at: https://www.ucl.ac.uk/global-health/research/z-research/tb-ipd-platform.

Research involving human participants, their data, or biological material

Policy information about studies with human participants or human data. See also policy information about sex, gender (identity/presentation), and sexual orientation and race, ethnicity and racism.

Reporting on sex and gender	Self reported Sex at birth, categorised as female or male, was used consistently throughout the study data collection as reported in table 1, and in the modelling.
Reporting on race, ethnicity, or other socially relevant groupings	Self reported race categorised as Asian, Black, Caucasian and Other, were collected for pharmacokinetic modelling based on prior evidence on differences in drug clearance (bedaquiline). Before integration the covariates were tested for collinearity
Population characteristics	As reported in table 1, these included age, sex, weight, body mass index (BMI), living with HIV infection and disease severity described by sputum smear grade and presence or absence of cavitation on chest x-ray
Recruitment	Participants in the investigational arms of the TB-PRACTECAL randomised controlled trial in Belarus and South Africa were offered to participate in the sub-study.
Ethics oversight	London School of Hygiene & Tropical Medicine Research Ethics Committee, the Médecins Sans Frontières Ethics Review Board. Site specific approvals were obtained from the Belarus Republican Scientific and Practical Centre of Pulmonology and Tuberculosis ethics committee for the Minsk site, PharmaEthics for the Don Mckenzie and Dorris Goodwin hospitals sites and the University of Witwatersrand Human Research ethics committee

Note that full information on the approval of the study protocol must also be provided in the manuscript.

Field-specific reporting

Please select the one below that is the best fit for your research. If you are not sure, read the appropriate sections before making your selection.

[x] Life sciences   [ ] Behavioural & social sciences   [ ] Ecological, evolutionary & environmental sciences

For a reference copy of the document with all sections, see nature.com/documents/nr-reporting-summary-flat.pdf

Life sciences study design

All studies must disclose on these points even when the disclosure is negative.

Sample size	No statistical method was used to predetermine sample size, however, during the optimal design process we used the Fisher Information Matrix to optimise a venous blood sampling design that supports pharmacokinetic model development which achieved the objectives at a sample size of 240 patients. We also recognised that as a sub-study, the actual sample size would be opportunistic.
Data exclusions	Results of drug concentration below the lower limit of quantification (defined as the lowest concentration for which the percentage deviation from the nominal standard concentration is less than 20 %) were excluded from analysis. As the proportion of LLQ for all drugs was below 10%, the M1 method was used.
Replication	Bioanalytical methods were validated by determination of the following parameters: Specificity and selectivity; recovery; stability; matrix effect; linearity; accuracy and precision; levels of detection and levels of quantification. validation was successful.
Randomization	This is not pertinent to this sub-study as recruitment was opportunistic, however in the main trial patients were randomly assigned in 1:1:1:1 ratio in stage 1 and in a 1:1 ratio in stage 2. Randomisation lists were prepared by trial statisticians and randomisation was stratified by study site.
Blinding	The main trial was open label but the mycobacteriology laboratory team were blinded to study allocation.

Reporting for specific materials, systems and methods

We require information from authors about some types of materials, experimental systems and methods used in many studies. Here, indicate whether each material, system or method listed is relevant to your study. If you are not sure if a list item applies to your research, read the appropriate section before selecting a response.

Materials & experimental systems

n/a	Involved in the study
☒	☐ Antibodies
☒	☐ Eukaryotic cell lines
☒	☐ Palaeontology and archaeology
☒	☐ Animals and other organisms
☐	☒ Clinical data
☒	☐ Dual use research of concern
☒	☐ Plants

Methods

n/a	Involved in the study
☒	☐ ChIP-seq
☒	☐ Flow cytometry
☒	☐ MRI-based neuroimaging

Clinical data

Policy information about clinical studies

All manuscripts should comply with the ICMJE guidelines for publication of clinical research and a completed CONSORT checklist must be included with all submissions.

Clinical trial registration: NCT04081077

Study protocol: Berry, C., du Cros, P., Fielding, K. et al. TB-PRACTECAL: study protocol for a randomised, controlled, open-label, phase II–III trial to evaluate the safety and efficacy of regimens containing bedaquiline and pretomanid for the treatment of adult patients with pulmonary multidrug-resistant tuberculosis. Trials 23, 484 (2022). https://doi.org/10.1186/s13063-022-06331-8 for the mycobacteriology, efficacy and safety.

Nyang'wa BT, Kloprogge F, Moore DAJ, Bustinduy A, Motta I, Berry C, Davies GR. Population pharmacokinetics and pharmacodynamics of investigational regimens' drugs in the TB-PRACTECAL clinical trial (the PRACTECAL-PKPD study): a prospective nested study protocol in a randomised controlled trial. BMJ Open. 2021 Sep 6;11(9):e047185. doi: 10.1136/bmjopen-2020-047185 for the PKPD

Data collection: Started in January 2017 for the main trial, the sub-study started in August 2019 and all recruitment was completed in March 2021 from four hospitals and health centres in Belarus, and South Africa. The last patient last visit was in september 2022

Outcomes: The key model derived outcomes were the area under the drug concentration curve (AUC), the lowest drug concentration just before the next dose (Ctrough), the highest drug concentration between two doses (Cmax). Together with cumulative percentage of the dosing interval that the drug concentration exceeds the MIC (T>MIC) drug exposure matrices were evaluated, adjusted for MIC or not, in the context of bacillary clearance.

The efficacy (second and third) objectives were to establish an exposure-response model for each drug and regimen to both early bactericidal activity and long-term treatment outcomes. The main endpoint was the change over time to the duration it took for a culture to have growth detected in MGIT, i.e. time to positivity (TTP).

The safety (fourth) objective was to investigate the exposure-toxicity relationship of each drug. The outcome measures for this analysis were adverse events, including elevated ALT, AST or bilirubin levels, decreased creatinine clearance, anaemia, neutropenia and peripheral neuropathy.

Plants

Seed stocks: *Report on the source of all seed stocks or other plant material used. If applicable, state the seed stock centre and catalogue number. If plant specimens were collected from the field, describe the collection location, date and sampling procedures.*

Novel plant genotypes: *Describe the methods by which all novel plant genotypes were produced. This includes those generated by transgenic approaches, gene editing, chemical/radiation-based mutagenesis and hybridization. For transgenic lines, describe the transformation method, the number of independent lines analyzed and the generation upon which experiments were performed. For gene-edited lines, describe the editor used, the endogenous sequence targeted for editing, the targeting guide RNA sequence (if applicable) and how the editor was applied.*

Authentication: *Describe any authentication procedures for each seed stock used or novel genotype generated. Describe any experiments used to assess the effect of a mutation and, where applicable, how potential secondary effects (e.g. second site T-DNA insertions, mosiacism, off-target gene editing) were examined.*
