# Supplementary material for: Pharmacokinetics, bactericidal activity and toxicity of short oral regimens for rifampicin-resistant tuberculosis treatment
Source: Nat Commun. 2026 Jun 10;17:7830. doi: 10.1038/s41467-026-74335-y (PMC13439552; doi:10.1038/s41467-026-74335-y)
Supplement: Supplementary file 3 [file 41467_2026_74335_MOESM3_ESM.pdf]

nature portfolio

Peer Review File

Pharmacokinetics, bactericidal activity and toxicity of short oral regimens for rifampicin-resistant tuberculosis treatment

Corresponding Author: Dr Bern Thomas Nyang'wa

This file contains all reviewer reports in order by version, followed by all author rebuttals in order by version.

Version 0:

Reviewer comments:

Reviewer #1

(Remarks to the Author)
Within the PRACTECAL trial, the authors prospectively designed a PK-PD sub study to explore exposure-response and exposure-toxicity relationships. This carefully designed PK-PD study sets an excellent precedent for the field. While challenging, such studies are sorely needed to establish PK-PD targets in the context of combination TB therapy. The authors are commanded for publishing their results despite the lack of association between exposure and response. This knowledge and its implications are important for the TB community.
The manuscript is clearly written and, while describing PK-PD models and modeling outcomes, remains approachable for the non-expert.
A noteworthy result is that severity of disease at treatment start has a more profound impact on outcome than drug exposure, at least in this cohort. This may be worth mentioning in the abstract.
From the Abstract: “Absence of correlation between drug exposure and bacillary clearance suggest that the dosages used achieve saturation of bacillary killing, while remaining safe.” is a strong statement for a regimen that requires 6 months to achieve cure. It seems just as likely that correlating exposure with response in the context of combination therapy would require larger cohorts and stratification between cavitary and non-cavitary. The conclusions drawn in lines 216-219 may be more appropriate for the abstract.
The methods are sufficiently detailed and clearly described, with one request: Table S2 needs additional legend/footnotes to describe the terms in column 1. Alternatively, please use full words (smear 1+, cavitary disease, etc…). Does “Mean estimate” refer to the slope? If yes, indicate that either in the column header or in the table title. What is the difference between Merged (BPaLCMerged) and not (BPaLM)?
Minor comments
Figure 4: some X axis labels run over each other, also please improve readability. Most fonts are too small
Figure S5 and S6: X axis labels are not readable

Reviewer #2

(Remarks to the Author)
Thank you for the opportunity to read and review the manuscript “Pharmacokinetics, bactericidal activity and toxicity of short oral regimens for rifampicin-resistant tuberculosis treatment” describing the partial result of a prospective pharmacokinetics and pharmacodynamics study nested in TB-PRACTECAL clinical trial. This is a well written manuscript, and I do not have any major comments on the results or methods used in this work.

A few suggestions, should the author want to consider, acknowledging the authors repeatedly cite previously published paper.

• It would be nice to add a few examples of the Standard-of-care at different study sites (Belarus and South Africa)or rifampin-resistant tuberculosis. This heterogeneity would probably highlight the further amplification of resistance based on different drug combination.

• Figure 2. Can the authors change the colors of the lines and thickness for different regimens? It would help some reviewers and reader like me (color blind).

• Lines 201-203. I would be careful with interpretation of dose adequacy for generalization. Although the authors acknowledged this in the very next sentence.

• Lines 215-217: arguing the studies dose of linezolid and moxifloxacin are safe. But are these doses optimal? Can you comment?

• Lines 265-269: The authors cite a previous publication for change in TTP and MIC. The present work being a PK/PD study and reporting exposures for efficacy and any associated toxicity will benefit from re-reporting those MIC changes and also an indication of acquired drug resistance if the MIC values increased over time.

• Line 303-305: How many patients relapsed?

Overall, a nicely done study and timely reporting of data.

Open Access This Peer Review File is licensed under a Creative Commons Attribution 4.0 International License, which permits use, sharing, adaptation, distribution and reproduction in any medium or format, as long as you give appropriate credit to the original author(s) and the source, provide a link to the Creative Commons license, and indicate if changes were made.
In cases where reviewers are anonymous, credit should be given to 'Anonymous Referee' and the source.
The images or other third party material in this Peer Review File are included in the article’s Creative Commons license, unless indicated otherwise in a credit line to the material. If material is not included in the article’s Creative Commons license and your intended use is not permitted by statutory regulation or exceeds the permitted use, you will need to obtain permission directly from the copyright holder.
To view a copy of this license, visit https://creativecommons.org/licenses/by/4.0/

REVIEWERS' COMMENTS

Reviewer #1 (Remarks to the Author)
Within the PRACTECAL trial, the authors prospectively designed a PK-PD sub study to explore exposure-response and exposure-toxicity relationships. This carefully designed PK-PD study sets an excellent precedent for the field. While challenging, such studies are sorely needed to establish PK-PD targets in the context of combination TB therapy. The authors are commanded for publishing their results despite the lack of association between exposure and response. This knowledge and its implications are important for the TB community.
The manuscript is clearly written and, while describing PK-PD models and modeling outcomes, remains approachable for the non-expert.

Thank you.

A noteworthy result is that severity of disease at treatment start has a more profound impact on outcome than drug exposure, at least in this cohort. This may be worth mentioning in the abstract.

Thank you, we added the following sentence in the abstract: “Also of note was a 20% decreased rate in patients with severe disease irrespective of treatment.”

From the Abstract: “Absence of correlation between drug exposure and bacillary clearance suggest that the dosages used achieve saturation of bacillary killing, while remaining safe.” is a strong statement for a regimen that requires 6 months to achieve cure. It seems just as likely that correlating exposure with response in the context of combination therapy would require larger cohorts and stratification between cavitary and non-cavitary. The conclusions drawn in lines 216-219 may be more appropriate for the abstract.

Thank you for the comment. We have amended the abstract conclusion to align with the main text.

The methods are sufficiently detailed and clearly described, with one request: Table S2 needs additional legend/footnotes to describe the terms in column 1. Alternatively, please use full words (smear 1+, cavitary disease, etc…). Does “Mean estimate” refer to the slope? If yes, indicate that either in the column header or in the table title. What is the difference between Merged (BPaLCMerged) and not (BPaLM)?

Thank you for the comment. We have updated the table with full words.

Minor comments
Figure 4: some X axis labels run over each other, also please improve readability. Most fonts are too small

Thank you. These have been corrected.

Figure S5 and S6: X axis labels are not readable
Thank you. These have been corrected.

Reviewer #2 (Remarks to the Author)
Thank you for the opportunity to read and review the manuscript "Pharmacokinetics, bactericidal activity and toxicity of short oral regimens for rifampicin-resistant tuberculosis treatment" describing the partial result of a prospective pharmacokinetics and pharmacodynamics study nested in TB-PRACTECAL clinical trial. This is a well written manuscript, and I do not have any major comments on the results or methods used in this work.

A few suggestions, should the author want to consider, acknowledging the authors repeatedly cite previously published paper.

• It would be nice to add a few examples of the Standard-of-care at different study sites (Belarus and South Africa)or rifampin-resistant tuberculosis. This heterogeneity would probably highlight the further amplification of resistance based on different drug combination.

Thank you for the suggestion, we have added a summary as follows: The standardised 9–11months regimens in Belarus consisted of high dose isoniazid, ethambutol, pyrazinamide, capreomycin, moxifloxacin, ethionamide and clofazimine (Hh/E/Z/Cm/Mfx/Eto/Cfz). In South Africa this was later replaced by bedaquiline, levofloxacin, linezolid, clofazimine and terizidone (B/Lfx/Lzd/Cfz/Trd). The longer 18-20 months regimen consisted of Z, Cm, Mfx, B, Lzd plus Eto, terizidone or PAS (Z/Cm/Mfx/B/Lzd plus Eto, Trd or PAS) and was individualised. From 2017 to 2019, these regimens generally included a second-line injectable agent and criteria for including bedaquiline were stringent. From 2019 to 2022, participants received all-oral versions of these regimens and most regimens included bedaquiline.

• Figure 2. Can the authors change the colors of the lines and thickness for different regimens? It would help some reviewers and reader like me (color blind).
Thank you for the recommendation, we have improved readability by increasing thickness of all lines and also differentiating the thickness of the unfavourable and favourable covariates.

• Lines 201-203. I would be careful with interpretation of dose adequacy for

generalization. Although the authors acknowledged this in the very next sentence.
Thank you for the comment, we indeed recognised that this could be one explanation but there could be others as well. We have changed the wording to emphasise this more and used “could suggest” instead.

• Lines 215-217: arguing the studies dose of linezolid and moxifloxacin are safe. But are these doses optimal? Can you comment?

Thank you for this important question. In our study, the association of haematological adverse events with higher linezolid exposure could suggest that higher doses could be detrimental without added benefit as postulated by the absence of an exposure-effect relationship. However, it is difficult to conclude if the linezolid dose was optimal through this study’s findings, although Kwak N. et al. (reference #8 in manuscript) using an individual patient data metanalysis suggested that in comparison to daily 600mg for 9 weeks, daily 600mg for 24 weeks, daily 1200mg for 24 weeks, 600mg for 16 weeks then 300mg for 8 weeks was better.

Moxifloxacin was used at the labelled dose, and Chirehwa *et al*. (2023) reported that an AUC of 26.5 mg*hr/l (which was the median in our study) is necessary to achieve an AUC/MIC PKPD target of 53 in the most prevalent MIC of 0.25mg/l. As there were no exposure-response trends for TTP (fig 3a) nor association with hepatic adverse events (fig 4) but faster clearance with BPaLM (fig 2), the 400mg dose could be considered optimal.

• Lines 265-269: The authors cite a previous publication for change in TTP and MIC. The present work being a PK/PD study and reporting exposures for efficacy and any associated toxicity will benefit from re-reporting those MIC changes and also an indication of acquired drug resistance if the MIC values increased over time.

Thank you for the request. We agree that increase in MIC over time may indicate acquired resistance. In that paragraph, we describe the findings from Nyang’wa *et al.(2024),* the parent randomised controlled trial where time to sputum culture conversion was reported. However, in this study we modelled the individual TTP data up to 12 weeks of treatment, at which point no changes in MIC were reported. The patients and mycobacteriology samples from these two studies are the same.

• Line 303-305: How many patients relapsed?
11/501 participants had recurrence at week108: five in BPaLC, four in the BPaL, one in the BPaLM, and one in the SOC.

Overall, a nicely done study and timely reporting of data.

Thank you.
